# Unveiling Antibiotic Resistance: Genome Sequencing of Streptomycin-Resistant *Erwinia amylovora* Isolate

**DOI:** 10.3390/microorganisms12122494

**Published:** 2024-12-03

**Authors:** Lin He, Yuna Kim, Seohyun Kim, Mi-Hyun Lee, Jun Myoung Yu

**Affiliations:** 1 Department of Applied Biology, Chungnam National University, Daejeon 34134, Republic of Korea; helin3022@gmail.com (L.H.); kyn5237@gmail.com (Y.K.); sobbed1213@gmail.com (S.K.); 2 Crop Protection Division, National Institute of Agricultural Sciences, Wanju 55365, Republic of Korea; mihyun798@korea.kr

**Keywords:** *Erwinia amylovora*, streptomycin resistance, whole genome, nonsynonymous single nucleotide variants (nsSNVs)

## Abstract

*Erwinia amylovora*, the causal agent of fire blight, poses a serious threat to several rosaceous plants, especially apples and pears. In this study, a spontaneous streptomycin-resistant *E. amylovora* strain (EaSmR) was isolated under laboratory conditions. Compared with the parental strain TS3128, the EaSmR strain exhibited high resistance to streptomycin (>100,000 µg/mL) and showed a significant reduction in both swimming and swarming motility. To investigate the mechanisms underlying streptomycin resistance, the genome of EaSmR was sequenced, and four single nucleotide variants (SNVs) were identified in comparison with the EaSmR genome with TS3128. Two genes in EaSmR were found to contain SNVs relative to TS3128, including a point mutation at codon 43 in the *rpsL* gene, the primary target of streptomycin, which was identified as the cause of the resistance. Additionally, three other point mutations were detected within the gene encoding type I methionyl aminopeptidase (MetAP1), resulting in an amino acid substitution from serine to valine (S76V). Furthermore, we analyzed the nonsynonymous single nucleotide variants (nsSNVs) between the EaSmR isolate and the reference type strain, CFBP1430. A total of 111 nsSNVs were found in EaSmR, including three stop-gain mutations, across 102 genes, which likely account for potential differences between the Korean strain TS3128 (EaSmR) and the reference strain CFBP1430. Whole-genome sequencing of EaSmR reveals significant genetic changes and provides valuable insights into the role of single nucleotide variants in antibiotic resistance and altered physiological traits. As the first report of a laboratory-induced, streptomycin-resistant *E. amylovora* strain from South Korea, this study provides essential insights into resistance mechanisms and highlights key genomic differences that may contribute to the unique characteristics of the Korean strain, establishing a valuable foundation for future disease management strategies.

## 1. Introduction

Fire blight, caused by *Erwinia amylovora*, is a devastating disease with a significant global economic impact on rosaceous plants, particularly apples and pears [1]. First identified as a bacterial disease in the 1880s, fire blight rapidly spread across numerous countries in North America, Europe, and New Zealand [2]. In Korea, fire blight was first reported in 2015 [3]. By 2021, the disease had spread to over 600 orchards in 28 cities, resulting in substantial economic losses [4,5,6]. In response to this crisis, the Korean government implemented several measures to control the spread of the disease. Aside from eradicating the diseased apple and pear trees, the management against fire blight primarily relied on chemical strategies based on copper compounds and antibiotics [7]. However, copper is only effective in controlling fire blight when applied before *E. amylovora* infects the blossoms, and it can also be harmful to developing fruit and foliage [8,9]. Antibiotics, particularly streptomycin, oxytetracycline, oxolinic acid, kasugamycin, and validamycin, are the most effective antimicrobial groups registered by the Rural Development Administration (RDA) of Korea for controlling fire blight in Korea [6,7,10]. Among these antibiotics, streptomycin-based antibiotics are favored for their high efficacy and relatively low cost [7,11].

While streptomycin effectively controls outbreaks of *E. amylovora*, streptomycin-resistant *E. amylovora* strains have emerged in several U.S. states, including California, Washington, Oregon, Missouri, Michigan, and New York [12,13,14,15]. Moreover, streptomycin-resistant strains have been reported in other regions, such as Israel [16], Mexico [17], New Zealand [18], and Egypt [19]. There are currently no reports of streptomycin-resistant *E. amylovora* in Korea. Two mechanisms of streptomycin resistance have been well studied in *E. amylovora*. One involves transferable resistance genes, which occur through the acquisition of the transposable element Tn5393, carried on plasmids such as pEA34 or pEA29. This element encodes two streptomycin modification enzymes via the gene pair *strA-strB*, leading to decreased susceptibility to streptomycin [20,21,22,23]. Alternatively, resistance can arise from a single base pair mutation in codon 43 of *rpsL*, which encodes the S12 protein of the 30S small ribosomal subunit. This mutation prevents streptomycin from binding to the ribosome while preserving the protein synthesis function [11,20].

In this study, a spontaneous streptomycin-resistant *E. amylovora* strain (EaSmR) was isolated from streptomycin-amended media under laboratory conditions. We characterized the EaSmR strain by determining its minimum inhibitory concentration for streptomycin, growth rate, motility, biofilm formation, and pathogenicity. Additionally, we analyzed the high-quality genome sequence of the EaSmR strain, which was assembled from PacBio and Illumina sequencing reads. Comparative analyses of single nucleotide variants (SNVs) were conducted between the EaSmR strain and both its parental strain TS3128 and the reference type strain CFBP1430, providing insights into the mechanisms underlying streptomycin resistance in the EaSmR strain and a deeper understanding of potential genetic differences between the Korean strain TS3128 and reference strain CFBP1430.

## 2. Materials and Methods

### 2.1. Isolation and Culture of the Strains

The parental strain of the EaSmR strain is *E. amylovora* TS3128, a virulent strain isolated from pear trees in Anseong, South Korea, in 2015, which serves as a reference strain for *E. amylovora* studies in Korea [24,25]. The streptomycin-resistant *E. amylovora* (EaSmR) colony was isolated from strain TS3128 cultured on Luria–Bertani (LB) agar supplemented with 100 µg/mL of streptomycin, demonstrating its ability to survive lethal concentrations of streptomycin. For purification, the EaSmR colony was streaked onto a fresh LB agar plate containing 100 µg/mL of streptomycin at 28 °C for two days. After incubation, single colonies of EaSmR were transferred to 10 mL of liquid LB and incubated at 28 °C with shaking at 180 rpm for 12 h for further study. To ensure biosafety and prevent exposure to the field, all experiments were conducted in a BSL-2 facility following appropriate protocols, and all materials used with EaSmR strains were autoclaved twice in leak-proof containers.

### 2.2. Biological Characterization of EaSmR

#### 2.2.1. Minimum Inhibitory Concentration

To determine the minimum inhibitory concentration (MIC) of streptomycin for the EaSmR strain, overnight cultures of TS3128 and EaSmR were adjusted to a final absorbance of 0.01 at OD600 in LB broth containing varying concentrations of streptomycin. The bacterial suspensions were dispensed into a 96-well plate at 100 µL per well. After 24 h of incubation at 28 °C with shaking at 250 rpm, the optical density at 600 nm (OD600) was measured to determine the antibiotic concentration that inhibited bacterial growth. An absorbance reading below 0.1 at 600 nm was considered to be indicative of growth inhibition. This experiment was conducted independently three times, with three technical replicates each.

#### 2.2.2. Growth Rate

To compare the growth of EaSmR and TS3128, overnight bacterial cultures were adjusted to an OD600 of 0.01 in LB medium. One-milliliter aliquots of each strain were separately transferred into 24-well plates and incubated at 28 °C with shaking at 250 rpm on a microplate shaker. The bacterial growth was measured at OD600 at 3 h intervals over a 24 h period.

#### 2.2.3. Motility Assays

Swimming, swarming, and twitching motility of TS3128 and EaSmR were examined following the protocols described by Yua, et al. [26] and Déziel et al. [27], with modifications. Swimming and swarming assays were performed on 90 mm LB agar plates containing 0.3% and 0.45% agar, respectively. For each assay, 10 μL of bacterial culture was spotted at the center of the plates. Twitching motility was assessed by stab-inoculating the bacteria onto LB plates containing 1% agar using a toothpick. The inoculated plates were incubated at 28 °C, and the diameters of the radial zones were measured to evaluate motility.

#### 2.2.4. Biofilm Formation

To compare the biofilm formation ability of TS3128 and EaSmR, a crystal violet staining method was conducted, as previously described in [28]. Briefly, overnight cultures of both strains were adjusted to an OD600 of 0.01. Then, 160 μL of the diluted cultures were transferred into 96-well plates and incubated at 28 °C for 48 h without shaking. After incubation, planktonic cells were removed by inverting the plate and tapping it on absorbent paper. The plates were then heat-fixed in a dry oven at 60 °C for 20 min. The wells were stained with 1% crystal violet for 20 min, rinsed with water, and air-dried. Finally, 220 μL of elution solution (40% ethanol and 10% acetone) was added to each well, and the OD600 of the solution was measured.

#### 2.2.5. Evaluation of Virulence

Before the bacterial inoculation, immature pear fruits (cv. Shin-go, Asian pear) were surface sterilized with 70% ethanol, rinsed with sterile distilled water, and allowed to air-dry completely. Overnight cultures of TS3128 and EaSmR were adjusted to a concentration of 10^8^ CFU/mL. Ten microliters of each bacterial suspension was inoculated into the fruit by creating approximately 2 mm incisions with pipette tips. To evaluate the symptom severity in the inoculated pears, a disease severity index (DSI) was developed based on the percentage of the average horizontal and vertical lesion diameters relative to the overall horizontal and vertical diameters of the fruit. The DSI was categorized into five levels, with each level representing a 20% increment in the infected area (characterized water-soaking and browning necrotic lesion): 0 (no lesion), 1 (0–20% infected area), 2 (20–40% infected area), 3 (40–60% infected area), 4 (60–80% infected area), and 5 (80–100% infected area).

### 2.3. Genomic DNA Extraction, Sequencing, Assembly, and Annotation

The genomic DNA of the EaSmR strain was extracted using a DNeasy Blood and Tissue Kit (QIAGEN, Valencia, CA, USA) by following the manufacturer’s instructions. The purity and concentration of the genomic DNA were evaluated using a NanoDrop UV–Vis Spectrophotometer (NanoPhotometer NP80, Implen, Munich, Germany). The integrity of the gDNA was assessed using agarose gel electrophoresis. The genomic sequencing of the EaSmR strain was executed via single-molecule real-time (SMRT) sequencing on the Pacific Biosciences Sequel platform and was complemented by library preparation utilizing the PacBio SMRTbell prep kit 3.0 (PacBio, Menlo Park, CA, USA). Illumina sequencing reads were obtained from the Illumina NovaSeq platform to enhance the contig assembly precision by employing a library prepared with the TruSeq Nano DNA Preparation Kit (Illumina, San Diego, CA, USA). These Illumina reads were instrumental in refining the assembly process, ensuring comprehensive coverage and fidelity in the genomic reconstruction. Sequencing was performed by Macrogen Co., Ltd. (Daejeon, Republic of Korea). De novo genome assembly of the EaSmR strain was performed using PacBio long reads with the Microbial Assembly application from SMRT Link v8.0 (https://www.pacb.com/) (accessed on 14 June 2022). Then, the Illumina reads were applied to the accurate genomic sequence using Pilon v1.21 [29]. For all the software used in this study, the default parameters were employed unless otherwise specified. The completeness of the assembled genome was evaluated using the Benchmarking Universal Single-Copy Orthologs (BUSCO, v5.1.3) dataset eukaryota_odb10 (Embryophyta) [30]. The genome sequence was annotated from CDSs, transfer (tRNA) genes, and ribosomal RNA (rRNA) genes using Prokka v1.13 [31]. Six databases were used to predict the gene functions. These included the GO database (https://geneontology.org/) (accessed on 14 June 2022), the InterPro (v69.0) database, the Pfam (v31.0) database, the CDD (v 3.16) database, the TIGRFAM (v15.0) database, and the EggNOG (v4.5) database. A blast search of the whole genome of the EaSmR strain was performed against the above six databases.

### 2.4. Analysis of Single Nucleotide Variants (nsSNVs)

To investigate the resistance mechanism, sequencing reads of the EaSmR strain were mapped to the genome of its parental strain, *E. amylovora* TS3128 (accession: GCA_013375015.1), using BWA-MEM [32]. High-confidence SNVs were filtered and identified with SAMtools (v0.1.16) [33]. The same approach was applied to generate SNVs by comparing the EaSmR strain to the reference genome *E. amylovora* CFBP1430 (accession: GCF_002952315.1). Functional annotation of the SNV-related genes was conducted using the Kyoto Encyclopedia of Genes and Genome (KEGG) database via the BLAST tool, and Cluster of Orthologous Groups (COG) annotation was performed with Eggnog-Mapper (http://eggnog-mapper.embl.de/) (accessed on 30 May 2024). 

### 2.5. Statistical Analysis

The significance of the data was statistically evaluated using Student’s *t*-test or two-way ANOVA with GraphPad Prism 9.5.1 (GraphPad Software, San Diego, CA, USA). Data are presented as mean values with standard error (SEM), and a *p*-value < 0.05 was considered statistically significant. All experiments were performed with three replications, with the details in the corresponding figure legends.

## 3. Results

### 3.1. Biological Characterization of Streptomycin-Resistant Strain EaSmR

**Figure 1:**
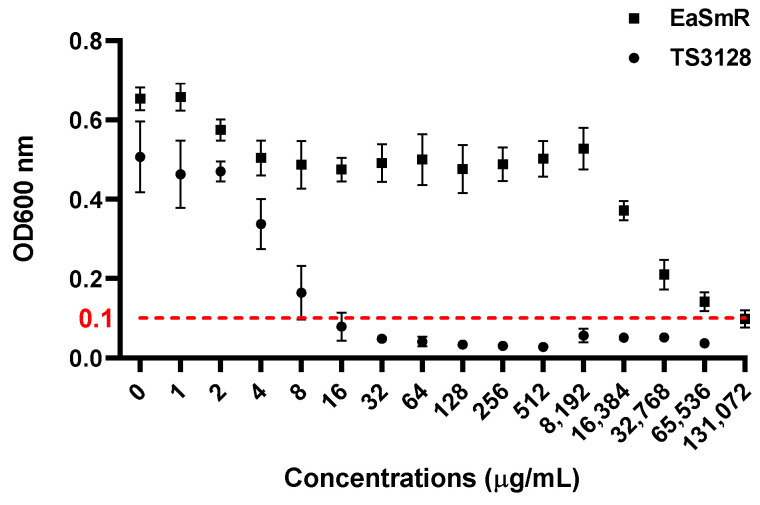
The minimum inhibitory concentration (MIC) of TS3128 (circle) and EaSmR (square) against streptomycin. The bacterial cultures were adjusted to a final absorbance of 0.01 at an OD 600 nm and exposed to streptomycin at varying concentrations. The MIC values were determined using 96-well plates incubated at 28 °C with 250 rpm for 24 h. The error bars represent the standard error of the mean (SEM) calculated from three replications, with a total sample size of *n* = 6 per group; the red dashed lines represent the OD cutoff for growth inhibition.

During the repeated sub-culturing of TS3128 on streptomycin-amended media, a spontaneous resistant strain, EaSmR, was isolated. To determine the streptomycin sensitivity of EaSmR, a minimum inhibitory concentration (MIC) assay was performed. As shown in Figure 1, the MIC of the streptomycin-sensitive TS3128 strain was 16 µg/mL. In contrast, the EaSmR strain exhibited a significantly higher resistance, with an MIC of up to 131,072 µg/mL under our conditions (Figure 1).

**Figure 2:**
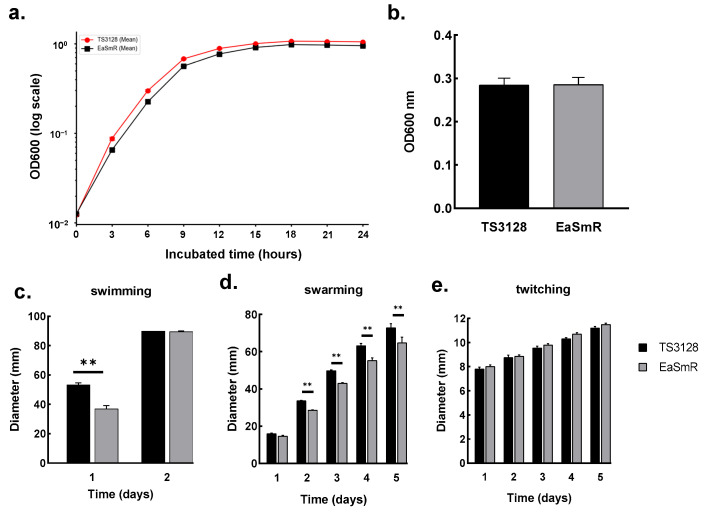
The biological characterization of the streptomycin-resistant strain EaSmR. (**a**) Growth curves of *Erwinia amylovora* TS3128 (circles) and EaSmR (squares) measured as OD600 over time in hours are presented on a semi-logarithmic scale. The data are the means of three biological replicates with three technical replicates for each; the error bars are smaller than the data labels. (**b**) Biofilm formation of *E. amylovora* TS3128 (black bar) and EaSmR (gray bar). The error bars indicate the standard error of the mean (SEM), *n* = 36 per group. No significance (ns) was detected using Student’s *t*-test. (**c**–**e**) The motility of *E. amylovora* TS3128 (black bars) and EaSmR (gray bars). (**c**) A swimming motility comparison; (**d**) swarming motility comparison; (**e**) twitching motility comparison. The error bars indicate the standard error of the mean (SEM). The experiment was performed with three biological and three technical replicates. The asterisks ** indicate statistically significant differences as determined by unpaired *t*-test (*p* < 0.05).

To compare the physiological characteristics of EaSmR, the growth curve, motility assays, and biofilm production were compared with those of TS3128. The results showed that both strains exhibited similar growth patterns, reaching the stationary phase after 18 h. However, EaSmR demonstrated slightly reduced population density compared with TS3128 at every stage of the growth curve (Figure 2a). In the biofilm assay, however, there were no significant differences in the biofilm formation capabilities between the two strains (Figure 2b).

The motility assays revealed that the EaSmR strain displayed a significantly reduced swimming ability compared to TS3128 on the first day. However, both strains, TS3128 and EaSmR, exhibited full growth on the plates after two days of inoculation in the swimming assay (Figure 2c). The swarming assay was conducted over a five-day period, and the results revealed that TS3128 exhibited a significantly faster swarming ability compared to the EaSmR isolate (Figure 2d). There was no discernible difference between the TS3128 and EaSmR strains in the twitching assay (Figure 2e).

**Figure 3:**
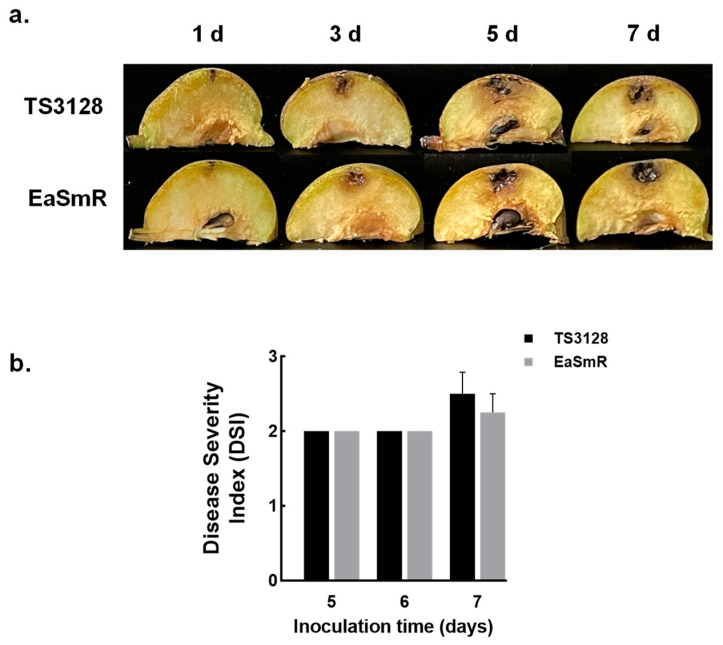
Comparison of virulence between *Erwinia amylovora* TS3128 and EaSmR in pear fruits. (**a**) A lateral section of the symptoms in immature pears inoculated with TS3128 or EaSmR. (**b**) The disease severity index (DSI) of pears injected with TS3128 (black bars) and EaSmR (gray bars) over 5–7 days. The DSI was evaluated on a scale comprising 0: no lesion; 1: 0–20% infected area; 2: 20–40% infected area; 3: 40–60% infected area; 4: 60–80% infected area; and 5: 80–100% infected area. The data are the means of three repeated experiments with four technical replicates (*n* = 12 of each group). The error bars indicate the standard error of the mean (SEM). Bonferroni’s multiple comparisons analysis showed no statistical difference in DSI between TS3128 and EaSmR.

For the virulence test, no significant differences were observed in disease development between the two strains following inoculation in immature pear fruits. Both strains resulted in water-soaked and browning necrotic symptoms on fruitlets three days post-inoculation. By days 5–7, the disease symptoms had worsened, with increased necrosis inside the fruits. Additionally, cavities formed within the infected fruits, accompanied by the accumulation of ooze (Figure 3a). In addition, the disease severity index (DSI) assay in fruitlets indicated no significant difference in the virulence between TS3128 and EaSmR (Figure 3b).

### 3.2. Genome Statistics of EaSmR

**Figure 4:**
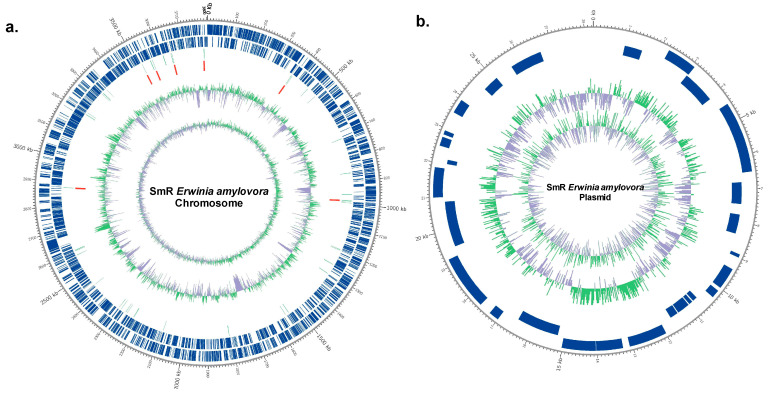
A circular representation of the genome of EaSmR. (**a**) Chromosome; (**b**) plasmid. From the outside to the inside of the circle graph are the predicted coding sequence (sense strand and antisense strand), transfer RNAs (tRNA), ribosomal RNAs (rRNA), GC content, and GC skew (indicated by the inner circle); positive (green), negative (purple).

A total of 91,629 subreads were generated from the PacBio platform sequencing, 830,030,786 bases. The sequencing achieved an N50 read length of 11,431 bp and a mean read length of 9058 bp, while the Illumina sequencing generated approximately 3646.2 Mb of raw data, including 2032.6 Mb of clean data (Table 1). The genome of EaSmR was found to be composed of one circular chromosome and one circular plasmid (Table 1; Figure 4). The chromosome was 3,804,100 bp in length with a GC content of 53.6%, while the plasmid was 28,251 bp in size with a GC content of 50.2%. The average sequencing depths for the chromosome and plasmid were 191.0× and 76.2×, respectively (Table 1). The assembled genome was 100% complete, with no fragments or missing data detected in the BUSCOs analysis. In the EaSmR genome, a total of 3401 protein-coding genes (CDSs) were predicted, with 3371 in the chromosome and 30 in the plasmid. Additionally, 78 tRNA genes and 22 rRNA genes were identified in the chromosome sequence (Table 1). The detailed features of the EaSmR strain are shown in Table 1. Among the protein-coding sequences, 2294 were mapped to the GO database (https://geneontology.org/) (accessed on 14 June 2022), 2825 proteins were characterized using the InterPro (v69.0) database, 2975 proteins were annotated via the Pfam (v31.0) database, 1045 proteins were identified through the CDD (v 3.16) database, 1325 proteins were assigned using the TIGRFAM (v15.0) database, and 3119 proteins were classified within the EggNOG (v4.5) database.

### 3.3. Genome Variants (nsSNVs)

The EaSmR isolate showed a high mapping rate of 100% to its parental strain TS3128 and 99.92% to the reference genome CFBP1430. Four SNVs were identified in the EaSmR isolate compared with the parental strain (TS3128) genome, with these nsSNVs distributed across two genes. One mutation is located in the *rpsL* gene at codon 43, encoding the S12 protein of the 30S small ribosomal subunit. This mutation involves a lysine-to-asparagine substitution (K43N), which alters the binding affinity of streptomycin at its target site, conferring streptomycin resistance to the bacteria. The other three mutations are found in the *map* gene, which encodes type I methionyl aminopeptidase (MetAP1), collectively resulting in a serine-to-valine substitution (S76V).

In the genome comparison between EaSmR and the reference genome CFBP1430, we focused on identifying nonsynonymous single nucleotide variants (nsSNVs) that cause amino acid changes within functional gene sequences as well as point mutations that result in premature termination codons (stop-gained mutations). The results showed that a total of 111 nsSNVs, including 3 stop-gained mutations, were identified in the EaSmR isolate compared to CFBP1430 distributed across 102 genes (Table S1). Notably, the same mutation in codon 43 of the *rpsL* gene was found in both EaSmR and CFBP1430, while no difference was detected in the *map* gene between the two genomes.

**Figure 5:**
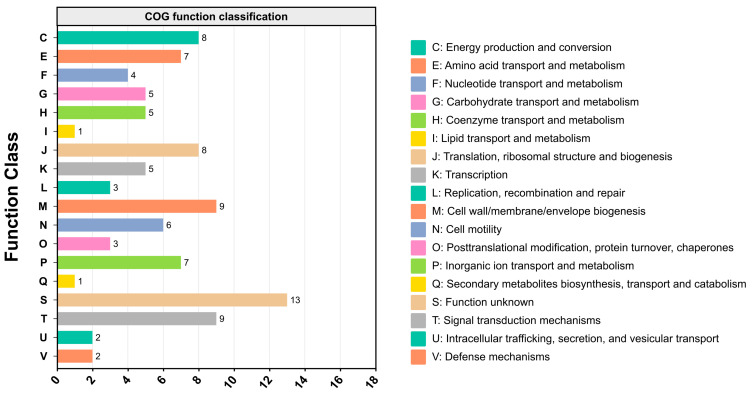
The COG functional distribution of genes containing nsSNVs between the EaSmR isolate and reference genome, CFBP1430.

In addition, nsSNVs were found within genes responsible for the multidrug efflux pump system (*acrB*, *yegN*, and *emrD*) (Table 2). In the EaSmR isolate, a pathogenicity-related gene, *invG,* was found to harbor nsSNVs (Table 2). Moreover, three stop-gained mutations of nsSNVs were located in the genes *EAMY_0860*, *tap*, and *tsr* (Table 2). Among these genes, *EAMY_0860* encodes the amino acid ABC transporter substrate-binding protein, and *tap* and *tsr* are related to the formation of methyl-accepting chemotaxis proteins, which contribute to cell motility. The COG analysis showed that the genes that carried nsSNVs were ascribed to 18 different functional classes. Many of the variant genes between the EaSmR isolate and reference strain CFBP1430 were predominantly associated with transport and metabolism (amino acid, nucleotide, carbohydrate, coenzyme, lipid, and inorganic ion; COG classifications in E, F, G, H, I, and P); energy production and conversion (C); translation, ribosomal structure, and biogenesis (J); cell wall/membrane/envelope biogenesis (M); signal transduction mechanisms (T); cell motility (N); and function unknown cluster groups (S) (Figure 5).

## 4. Discussion

The apple and pear industry in Korea faces significant challenges due to the widespread occurrence of fire blight caused by *E. amylovora*. In the laboratory environment, we isolated a spontaneous EaSmR strain capable of surviving high concentrations of streptomycin (Figure 1), which raises serious concerns given that streptomycin is a primary antibiotic widely used in Korea for fire blight control. In the current Korean fire blight management system, antibiotics containing streptomycin and oxytetracycline are primarily applied at 5 and 15 days after full bloom [34]. Although extensive screenings for streptomycin resistance have been conducted, there have been no reports to date of naturally occurring streptomycin-resistant *E. amylovora* in Korea [35].

Whole-genome sequencing (WGS) methods provide rapid and accurate sequence information, making it a powerful tool for investigating antibiotic resistance and physiological traits. In this study, we utilized WGS to explore the genomic variations and mechanisms associated with streptomycin resistance in the EaSmR isolate. Our analysis identified two mutated genes, *rpsL* and *map*, in EaSmR compared with its parental strain, TS3128. Notably, the *map* gene in EaSmR was identical to that in the reference genome of CFBP1430. Further BLAST analysis in the NCBI database revealed that, except for TS3128, the *map* gene sequence is highly conserved across other *E. amylovora* strains. This suggests that the differences observed in the TS3128 *map* gene sequence may result from sequencing artifacts or unique mutations specific to this strain. Additionally, a key nonsynonymous mutation in the *rpsL* gene of EaSmR, when compared with both TS3128 and CFBP1430, modifies streptomycin’s binding affinity at its target site. This mutation represents a well-known, established mechanism for high-level streptomycin resistance [23,36,37], explaining the resistance phenotype observed in EaSmR.

Apart from streptomycin resistance, the EaSmR isolate exhibited slightly reduced population density at each growth state (Figure 2a), as well as swimming and swarming motility compared to its parental strain TS3128 (Figure 2c,d). These changes likely indicate a “resistance cost”, a common phenomenon associated with chromosomal mutations conferring resistance or with mobile genetic elements carrying resistance genes [38]. Chromosomal mutations often disrupt essential cellular processes targeted by antibiotics, such as transcription, translation, or cell wall synthesis [38]. In the absence of selective antibiotic pressure, these mutations typically reduce the fitness of the microorganism, impacting its growth and overall vitality [39]. The *rpsL* gene, which encodes the S12 protein of the bacterial ribosome, is a key component of the 30S ribosomal subunit and plays a vital role in protein synthesis. It contributes to the structural stability and functionality of the ribosome, particularly in the binding of mRNA and tRNA. Furthermore, the RpsL protein also serves as a target for aminoglycoside antibiotics, including streptomycin and paromomycin [40]. Thus, mutations in the *rpsL* gene can affect translation accuracy and bacterial growth. A promising direction for future research would be to use site-directed mutagenesis to reverse the mutation in the *rpsL* gene. This approach could yield valuable insights into the mechanisms behind streptomycin resistance and further elucidate the connection between mutation-induced resistance and phenotypic changes in bacterial fitness.

In the present study, a genome comparison was also conducted between the EaSmR isolate and the reference genome, CFBP1430, revealing several notable nsSNV-associated genes (Table 2). Among these genes, three genes—*acrB*, *yegN*, and *emrD*—encode multidrug efflux pumps, which can export multiple antibiotics from the bacterial cells [41,42,43,44]. Additionally, two stop-gained nsSNV mutations were identified in the *tap* and *tsr* genes of the EaSmR isolate. Both of these genes play a role in the formation of methyl-accepting chemotaxis proteins (MCPs), which act as chemoreceptors in bacteria. MCPs detect intercellular and environmental signals, facilitating signal transmission to downstream pathways within the cytoplasm. They are crucial for biofilm formation, degradation of xenobiotic compounds, flagellum biosynthesis, toxin production, and exopolysaccharide production [45,46,47]. Moreover, an nsSNV was identified in a pathogenicity-related gene, *invG*, in the EaSmR isolate. This gene, part of the type III secretion system, encodes homologs of the PulD protein family, which is essential for secreting effector proteins into host cells. The InvG protein plays a key role in initial host cell binding, a critical step in the bacterial infection process [48].

To illustrate genome-wide differences in nsSNV-associated genes, a COG analysis was performed, revealing that these genes predominantly fall within functional groups linked to transport and metabolism, energy production and conversion, translation and ribosome biogenesis, cell wall/membrane biogenesis, signal transduction, and cell motility. Variations in genes associated with transport and metabolism may influence metabolic efficiency and adaptability [49]; differences in energy metabolism genes could result in altered energy requirements [50]; mutations in ribosomal genes may impact protein synthesis efficiency [51]; changes in structural genes are linked to antibiotic resistance and environmental adaptability [52]; alterations in signal transduction pathways might modify bacterial responses to environmental stimuli [53]; and mutations in motility genes could affect bacterial motility and colonization potential [54]. In summary, the genetic differences between the Korean isolate strain TS3128 (including EaSmR) and the reference strain CFBP1430 suggest potential variations in bacterial resistance, environmental adaptability, and cellular behavior likely driven by adaptive evolution, selective pressures, environmental factors, and distinct energy requirements.

In this study, whole-genome sequencing (WGS) provided critical insights into the mechanisms of streptomycin resistance and the physiological alterations observed in the EaSmR strain. The nsSNV analysis comparing TS3128 (EaSmR) with the reference strain CFBP1430 establishes a preliminary foundation for hypothesizing the physiological differences between the Korean *E. amylovora* isolate TS3128 and CFBP1430, though further experimental validation is needed. This genomic comparison highlights potential factors that may contribute to the unique traits of the TS3128 strain, offering a basis for future research into the adaptive responses and functional variations in *E. amylovora* strains from different regions.

## 5. Conclusions

A spontaneous streptomycin-resistant strain of *E. amylovora* was isolated under laboratory conditions. In this study, we compared several biological characteristics, including growth rate, motility, biofilm formation, and virulence, between the parental strain TS3128 and EaSmR. Additionally, we generated a high-quality genome assembly of the EaSmR isolate. Through comparative analysis, we identified single nucleotide variants (SNVs) in the EaSmR strain compared with its parental strain (TS3128) and the reference genome (CFBP1430), providing insights into the mechanisms of streptomycin resistance and highlighting potential differences between the Korean *E. amylovora* isolate and the reference strain. This study, as the first to report a laboratory-induced, streptomycin-resistant *E. amylovora* strain from South Korea, offers key insights into resistance mechanisms and genomic differences that may contribute to the unique characteristics of the Korean strain. These findings lay the groundwork for future disease management strategies.

## Figures and Tables

**Table 1:** Genome statistics of EaSmR.

Features	Statistics	
**PacBio Sequel platform**		
Number of reads	91,629	
Total length of reads	830,030,786 bp	
N50 length	11,431 bp	
Average read length	9058 bp	
**Illumina NovaSeq platform**		
Raw data of length	3,674,192,098 bp	
Clean data of length	2,032,571,077 bp	
Raw data of read	24,332,398	
Clean data of read	13,466,294	
Clean data Q20	99.06%	
Clean data Q30	96.0%	
Clean data GC	54.35%	
**Genome assembly**	Chromosome	Plasmid
Contig number	1	1
Circular	YES	YES
Contig size	3,804,100 bp	28,251 bp
GC content	53.6%	50.2%
Depth	191.0×	76.2×
**Genome annotation**		
Putative protein-coding genes	3371	30
Number of tRNA	78	0
Number of rRNA	22	0
**BUSCOs**		
Complete and single-copy BUSCOs	124 (100%)	
Complete and duplicated BUSCOs	0 (0.00%)	
Fragmented BUSCOs	0 (0.00%)	
Missing BUSCOs	0 (0.00%)	
Total BUSCO groups searched	124 (100%)	

**Table 2:** Key nonsynonymous single nucleotide variants (nsSNVs) identified in the EaSmR isolate compared with the reference CFBP1430 genome.

Gene ID	Name	Function Description	COG Cluster	Position in CDS	Amino Acid Changed
EAMY_3391	*rpsL*	30S ribosomal protein S12	J	129	K(AAA)→N(AAC)
EAMY_0123	*emrD*	multidrug transporter EmrD	E, G, P	587	S(TCT)→F(TTT)
EAMY_1008	*acrB*	multidrug efflux RND transporter permease subunit	V	932	V(GTT)→G(GGT)
EAMY_2263	*yegN*	multidrug transporter subunit MdtB	V	2573	A(GCC)→V(GTC)
EAMY_0777	*invG*	EscC/YscC/HrcC family type III secretion system outer membrane ring protein	N, U	71	S(AGC)→I(ATC)
EAMY_2090	*tap*	methyl-accepting chemotaxis protein	N, T	832	Q(CAG)→* (TAG) (Stop gained)
EAMY_2657	*tsr*	methyl-accepting chemotaxis protein	N, T	1579	Q(CAG)→* (TAG) (Stop gained)

The asterisk (*) represents the termination codon.

## Data Availability

The genome sequence in this article was deposited at DDBJ/ENA/GenBank under accession numbers CP171267 for chromosome and CP171268 for plasmid. The versions described in this study are CP171267 (chromosome) and CP171268 (plasmid) under the BioProject: PRJNA1168276 and BioSample: SAMN44032744.

## Supplementary Materials

The following supporting information can be downloaded at: https://www.mdpi.com/article/10.3390/microorganisms12122494/s1, Table S1. A list of all the identified nonsynonymous single nucleotide variants (nsSNVs) in the streptomycin-resistant *E. amylovora* strain (EaSmR) compared with the CFBP1430 reference genome.

## References

[1] Vanneste J.L. (2000). Fire Blight: The Disease and Its Causative Agent, Erwinia amylovora.

[2] Gayder S., Parcey M., Castle A.J., Svircev A.M. (2019). Host range of bacteriophages against a world-wide collection of *Erwinia amylovora* determined using a quantitative PCR assay. Viruses.

[3] Park D., Yu J., Oh E., Han K., Yea M., Lee S., Myung I., Shim H., Oh C. (2016). First report of fire blight disease on Asian pear caused by *Erwinia amylovora* in Korea. Plant Dis..

[4] Ham H., Lee Y.K., Kong H.G., Hong S.J., Lee K.J., Oh G.R., Lee M.H., Lee Y.H. (2020). Outbreak of fire blight of apple and Asian pear in 2015–2019 in Korea. Res. Plant Dis..

[5] Jik Lee H., Woo Lee S., Suh S.J., Hyun I.H. (2022). Recent spread and potential pathways for fire blight in South Korea. EPPO Bull..

[6] Choi J.H., Kim J.Y., Park D.H. (2022). Evidence of greater competitive fitness of *Erwinia amylovora* over *E. pyrifoliae* in Korean isolates. Plant Pathol. J..

[7] Kim Y.J., Choi H.S., Park D.H. (2024). Persistence and viable but non-culturable state induced by streptomycin in *Erwinia amylovora*. Front. Microbiol..

[8] Jamar L., Lateur M. Strategies to reduce copper use in organic apple production. Proceedings of the I International Symposium on Organic Apple and Pear 737.

[9] Montag J., Schreiber L., Schönherr J. (2006). An in vitro study of the nature of protective activities of copper sulphate, copper hydroxide and copper oxide against conidia of *Venturia inaequalis*. J. Phytopathol..

[10] Park D., Lee Y., Kim J., Cha J., Oh C. (2017). Current status of fire blight caused by *Erwinia amylovora* and action for its management in Korea. J. Plant Pathol..

[11] McManus P.S., Stockwell V.O., Sundin G.W., Jones A.L. (2002). Antibiotic use in plant agriculture. Annu. Rev. Phytopathol..

[12] Jimenez Madrid A.M., Ivey M.L.L. (2023). An overview of streptomycin resistance in *Erwinia amylovora* from Ohio apple orchards. Plant Health Prog..

[13] Tancos K., Villani S., Kuehne S., Borejsza Wysocka E., Breth D., Carol J., Aldwinckle H., Cox K. (2016). Prevalence of streptomycin-resistant *Erwinia amylovora* in New York apple orchards. Plant Dis..

[14] Miller T., Schroth M. (1972). Monitoring the epiphytic population of *Erwinia amylovora*. Phytopathology.

[15] Coyier D., Covey R. (1975). Tolerance of *Erwinia amylovora* to streptomycin sulfate in Oregon and Washington. Plant Dis. Rep..

[16] Manulis S., Zutra D., Kleitman F., Dror O., David I., Zilberstaine M., Shabi E. (1998). Distribution of streptomycin-resistant strains of *Erwinia amylovora* in Israel and occurrence of blossom blight in the autumn. Phytoparasitica.

[17] de León Door A.P., Romo Chacón A., Acosta Muñiz C. (2013). Detection of streptomycin resistance in *Erwinia amylovora* strains isolated from apple orchards in Chihuahua, Mexico. Eur. J. Plant Pathol..

[18] Thomson S., Gouk S., Vanneste J., Hale C., Clark R. The presence of streptomycin resistant strains of *Erwinia amylovora* in New Zealand. Proceedings of the VI International Workshop on Fire Blight 338.

[19] El Goorani M., El Kasheir H., Shoeib A.A., Hassanein F.M. (1989). Distribution of streptomycin resistant strains of *Erwinia amylovora* in Egypt during 1988. J. Phytopathol..

[20] Chiou C.S., Jones A. (1995). Molecular analysis of high-level streptomycin resistance in *Erwinia amylovora*. Phytopathology.

[21] Rezzonico F., Stockwell V.O., Duffy B. (2009). Plant agricultural streptomycin formulations do not carry antibiotic resistance genes. Antimicrob. Agents Chemother..

[22] McGhee G.C., Sundin G.W. (2012). *Erwinia amylovora* CRISPR elements provide new tools for evaluating strain diversity and for microbial source tracking. PLoS ONE.

[23] Escursell M.M., Roschi A., Smits T.H., Rezzonico F. (2021). Characterization and direct molecular discrimination of *rpsL* mutations leading to high streptomycin resistance in *Erwinia amylovora*. J. Plant Pathol..

[24] Kang I.J., Park D.H., Lee Y.K., Han S.W., Kwak Y.S., Oh C.S. (2021). Complete genome sequence of *Erwinia amylovora* strain TS3128, a Korean strain isolated in an Asian pear orchard in 2015. Microbiol. Resour. Ann..

[25] Myung I., Lee J., Yun M., Lee Y., Lee Y., Park D., Oh C. (2016). Fire blight of apple, caused by *Erwinia amylovora*, a new disease in Korea. Plant Dis..

[26] Yuan X., Eldred L.I., Sundin G.W. (2022). Exopolysaccharides amylovoran and levan contribute to sliding motility in the fire blight pathogen *Erwinia amylovora*. Environ. Microbiol..

[27] Déziel E., Comeau Y., Villemur R. (2001). Initiation of biofilm formation by *Pseudomonas aeruginosa* 57RP correlates with emergence of hyperpiliated and highly adherent phenotypic variants deficient in swimming, swarming, and twitching motilities. J. Bacteriol..

[28] O‘Toole G.A., Pratt L.A., Watnick P.I., Newman D.K., Weaver V.B., Kolter R. (1999). [6] Genetic approaches to study of biofilms. Methods Enzymol..

[29] Walker B.J., Abeel T., Shea T., Priest M., Abouelliel A., Sakthikumar S., Cuomo C.A., Zeng Q., Wortman J., Young S.K. (2014). Pilon: An integrated tool for comprehensive microbial variant detection and genome assembly improvement. PLoS ONE.

[30] Seppey M., Manni M., Zdobnov E.M. (2019). BUSCO: Assessing Genome Assembly and Annotation Completeness.

[31] Seemann T. (2014). Prokka: Rapid prokaryotic genome annotation. Bioinformatics.

[32] Li H. (2013). Aligning sequence reads, clone sequences and assembly contigs with BWA-MEM. arXiv.

[33] Li H., Handsaker B., Wysoker A., Fennell T., Ruan J., Homer N., Marth G., Abecasis G., Durbin R. (2009). The sequence alignment/map format and SAMtools. Bioinformatics.

[34] Ham H., Lee K.J., Hong S.J., Kong H.G., Lee M.-H., Kim H.-R., Lee Y.H. (2020). Outbreak of fire blight of apple and pear and its characteristics in Korea in 2019. Res. Plant Dis..

[35] Ham H., Oh G.-R., Lee B.W., Lee Y.H., Lee Y.H. (2024). Changes of sensitivity to streptomycin in Erwinia amylovora isolated from 2019 to 2023 in Korea. Res. Plant Dis..

[36] Nair J., Rouse D.A., Bai G.H., Morris S.L. (1993). The rpsL gene and streptomycin resistance in single and multiple drug-resistant strains of Mycobacterium tuberculosis. Mol. Microbiol..

[37] Springer B., Kidan Y.G., Prammananan T., Ellrott K., Böttger E.C., Sander P. (2001). Mechanisms of streptomycin resistance: Selection of mutations in the 16S rRNA gene conferring resistance. Antimicrob. Agents Chemother..

[38] Durão P., Balbontín R., Gordo I. (2018). Evolutionary mechanisms shaping the maintenance of antibiotic resistance. Trends Microbiol..

[39] Levin B.R., Perrot V., Walker N. (2000). Compensatory mutations, antibiotic resistance and the population genetics of adaptive evolution in bacteria. Genetics.

[40] Koshla O., Lopatniuk M., Borys O., Misaki Y., Kravets V., Ostash I., Shemediuk A., Ochi K., Luzhetskyy A., Fedorenko V. (2021). Genetically engineered *rpsL* merodiploidy impacts secondary metabolism and antibiotic resistance in Streptomyces. World J. Microbiol. Biotechnol..

[41] Nishino K., Yamasaki S., Nakashima R., Zwama M., Hayashi-Nishino M. (2021). Function and inhibitory mechanisms of multidrug efflux pumps. Front. Microbiol..

[42] Sjuts H., Vargiu A.V., Kwasny S.M., Nguyen S.T., Kim H.S., Ding X., Ornik A.R., Ruggerone P., Bowlin T.L., Nikaido H. (2016). Molecular basis for inhibition of AcrB multidrug efflux pump by novel and powerful pyranopyridine derivatives. Proc. Natl. Acad. Sci. USA.

[43] Nishino K., Yamaguchi A. (2001). Analysis of a complete library of putative drug transporter genes in *Escherichia coli*. J. Bacteriol..

[44] Baker J., Wright S.H., Tama F. (2012). Simulations of substrate transport in the multidrug transporter EmrD. Proteins.

[45] Salah Ud-Din A.I.M., Roujeinikova A. (2017). Methyl-accepting chemotaxis proteins: A core sensing element in prokaryotes and archaea. Cell. Mol. Life Sci..

[46] He K., Bauer C.E. (2014). Chemosensory signaling systems that control bacterial survival. Trends Microbiol..

[47] Black W.P., Yang Z. (2004). *Myxococcus Xanthus* chemotaxis homologs DifD and DifG negatively regulate fibril polysaccharide production. J. Bacteriol..

[48] Kaniga K., Bossio J.C., Galán J.E. (1994). The Salmonella typhimurium invasion genes invF and invG encode homologues of the AraC and PulD family of proteins. Mol. Microbiol..

[49] Västermark Å., Saier M.H. (2014). The involvement of transport proteins in transcriptional and metabolic regulation. Curr. Opin. Microbiol..

[50] Ward B. (2015). Bacterial energy metabolism. Molecular Medical Microbiology.

[51] Seip B., Innis C.A. (2016). How widespread is metabolite sensing by ribosome-arresting nascent peptides?. J. Mol. Biol..

[52] Jansen G., Mahrt N., Tueffers L., Barbosa C., Harjes M., Adolph G., Friedrichs A., Krenz-Weinreich A., Rosenstiel P., Schulenburg H. (2016). Association between clinical antibiotic resistance and susceptibility of *Pseudomonas* in the cystic fibrosis lung. Evol. Med. Public Health.

[53] Baker M.D., Wolanin P.M., Stock J.B. (2006). Signal transduction in bacterial chemotaxis. Bioessays.

[54] Colin R., Ni B., Laganenka L., Sourjik V. (2021). Multiple functions of flagellar motility and chemotaxis in bacterial physiology. FEMS Microbiol. Rev..

